# Monitoring protein-metal binding by ^19^F NMR – a case study with the New Delhi metallo-β-lactamase 1†
†Electronic supplementary information (ESI) available. See DOI: 10.1039/c9md00416e


**DOI:** 10.1039/c9md00416e

**Published:** 2020-02-21

**Authors:** Anna M. Rydzik, Jürgen Brem, Shane A. Chandler, Justin L. P. Benesch, Timothy D. W. Claridge, Christopher J. Schofield

**Affiliations:** a The Department of Chemistry , University of Oxford , 12 Mansfield Road , Oxford , OX1 3TA , UK . Email: christopher.schofield@chem.ox.a.uk

## Abstract

^19^F NMR protein observed spectroscopy is evaluated as a method for analysing protein metal binding using the New Delhi metallo-β-lactamase 1. The results imply ^19^F NMR is useful for analysis of different metallated protein states and investigations on equilibrium states in the presence of inhibitors. One limitation is that ^19^F labelling may affect metal ion binding. The sensitive readout of changes in protein behaviour observed by ^19^F NMR spectra coupled with the broad scope of tolerated conditions (*e.g.* buffer variations) means ^19^F NMR should be further investigated for studying metal ion interactions and the inhibition of metallo-enzymes during drug discovery.

## Abstract 2

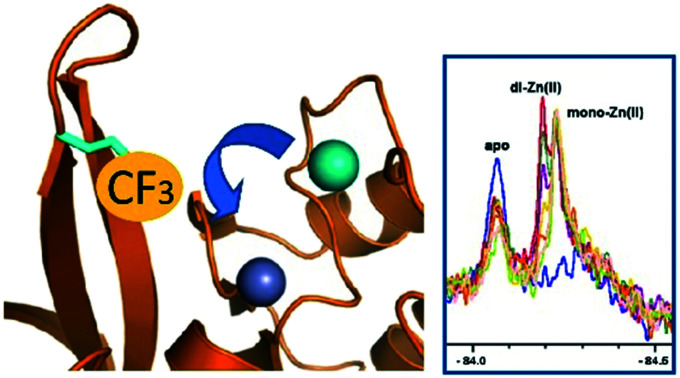
 ^19^F protein labeling enables monitoring of metal binding to the active site of NDM-1.

Although discovered >7 decades ago, the β-lactams remain amongst the most widely used antibiotics.^3^–^5^ Bacteria have evolved multiple resistance mechanisms to β-lactams, of which the best characterised involves β-lactamase catalysis (Fig. 1A).^3^ Although the nucleophilic serine-β-lactamases are most widespread, metallo-β-lactamases (MBLs) are of increasing clinical relevance.^4^ Amongst MBLs the New Delhi metallo-β-lactamase 1 (NDM-1) is of concern because of its distribution and ability to confer resistance to even the latest generations of β-lactam antibiotics/serine β-lactamase inhibitors.^5^–^7^ Mechanistic and structural studies of MBLs are relevant to the development of inhibitors aimed at preserving and extending β-lactam efficacy.^8^

**Fig. 1:**
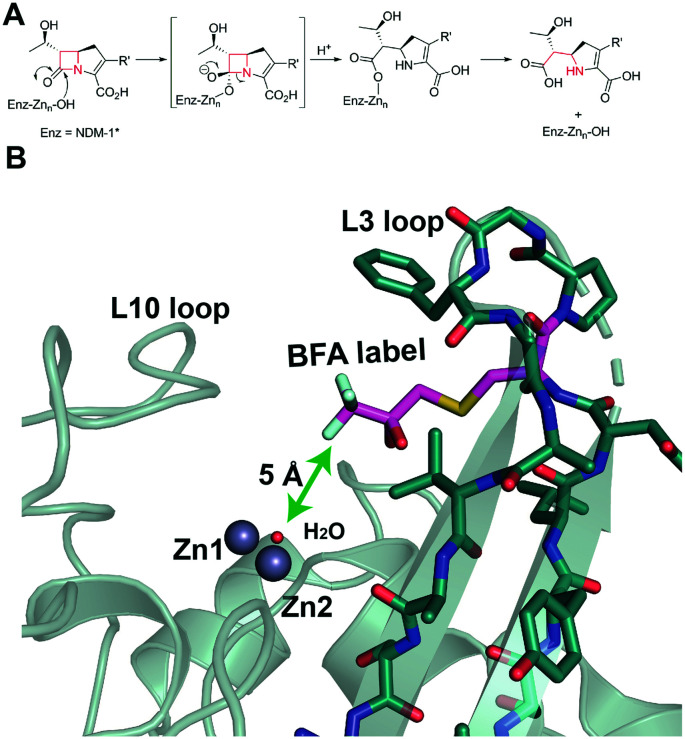
Metallo β-lactamase catalysis and site of ^19^F labelling. (A) Outline mechanism of B1 MBLs. (B) Location of the trifluoroketone label (Enz-Cys (67)-SCH_2_C(OH)_2_CF_3_) based on a wildtype NDM-1 crystal structure (PDB ID: ; 5ZGW).^1^ Note the trifluoroketone may be hydrated and is in a conformationally flexible region.^2^

MBLs are divided into 3 subclasses (B1, B2, B3), based on sequence and structural similarities, with the B1 MBLs presently being the most clinically relevant.^6^ Most B1 MBLs, including NDM-1, are proposed to utilise two closely bound Zn(ii) ions (Zn1/Zn2) with a bridging hydroxide/water.^8^–^11^ The two MBL metal ion binding sites have distinctive coordination chemistry, *i.e.* the Zn1 (or 3H) site employs H116, H118, and H196 as ligands and the Zn2 (or DCH) site employs D120, C221, and H263 as ligands.^8^ In a cellular context, MBLs are proposed to employ Zn(ii) ions; however, other metal ions support catalysis by isolated NDM-1 (ref. 12 and 13) and NDM-variants can manifest different metal ion stoichiometry.^8^,^10^ The determination of precise protein metallation states in solution is often non-trivial and has led to uncertainties, including in the MBL field, with respect to MBL:metal ion stoichiometry^9^ and the number of metal ions necessary for catalysis.^10^,^13^

We are interested in developing solution based NMR methods for monitoring binding of ligands and metal ions to metallo-proteins.^11^,^14^–^16^ Protein-observed ^19^F NMR is being increasingly used in biophysical analyses and drug discovery.^17^,^18^ This is due to the high natural abundance of ^19^F (spin quantum number 1/2), the 83% signal sensitivity of ^19^F relative to ^1^H NMR, the sensitivity of ^19^F chemical shifts to environment,^18^,^19^ and advances in ^19^F labelling. We have reported that introduction of a ^19^F label into NDM-1 near its active site (by alkylation of M67C NDM-1) enables monitoring of ligand binding by ^19^F NMR (Fig. 1B).^20^ We were thus interested to investigate if ^19^F NMR could be used for studying metal ion binding to NDM-1.

As reported, NDM-1 was labelled on the active site bordering L3 loop by efficient alkylation of NDM-1 M67C by 3-bromo-1,1,1-trifluoroacetone (BFA) to yield singly trifluoromethylated NDM-1 (NDM-1*), with small changes in catalytic properties (Table S1†).^20^ We initially investigated binding of Zn(ii) and Cd(ii) ions to apo NDM-1* using ^19^F NMR (Fig. 2 and S1–8†). Apo-NDM-1* manifests two main sets of ^19^F resonances (I and II, Fig. 2), potentially reflecting two sets of conformational isomers in a state of slow exchange.^20^ Upon Zn(ii) ion titration, significantly different ^19^F NMR signals were observed (Fig. 2A and S1,† III and IV, with the latter at lower shift). The ^19^F resonances III and IV (Fig. 2A and S1†) potentially correspond to the di- and mono-Zn(ii) bound species, respectively, since III dominates at high Zn(ii) and the intensity of IV decreases with increasing Zn(ii) (Fig. 2A and S1†). These assignments are supported by non-denaturing MS observations, which show both mono- and di-Zn(ii) metallated complexes of NDM-1 can co-exist (Fig. S10 and S11†).

**Fig. 2:**
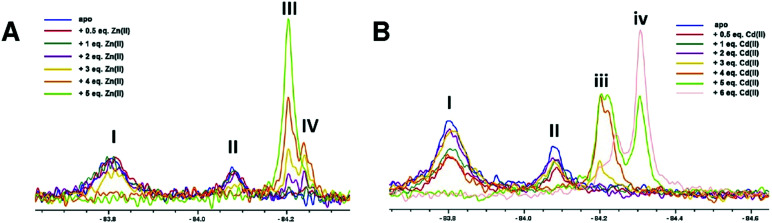
^19^F NMR analysis of the titration of ^19^F labelled apo-NDM-1* with (A) Zn(ii) and (B) Cd(ii) ions, at pH 7.5. (A) Peaks I and II correspond to apo-NDM-1*. III increases and IV decreases with increasing Zn(ii) ion ions, suggesting they reflect di- and mono-Zn(ii) forms, respectively. In the case of increasing Cd(ii) ions, iii decreases and iv increases, suggesting they reflect mono and di-Cd(ii) forms, respectively.

With Cd(ii) ion titration, broadly analogous results were obtained, though this time the more shielded signal (iv) dominates at higher metal ion (Cd(ii)) concentration, suggesting this likely corresponds to the di-Cd(ii) species (Fig. 2B and S5†). Notably, even with excess Cd(ii), signals in the region of (iii) and a signal intermediate between (iii) and (iv) were observed (Fig. 2B). These observations are proposed to reflect weaker binding of a second Cd(ii) ion compared to a second Zn(ii) ion, as reported for some MBLs (consistent with the larger ionic radius of Cd(ii) compared with Zn(ii)).^13^,^21^,^22^ It should also be noted that the kinetics of MBL catalysis and inhibition can change depending on the active site metal ions present (*e.g.* Zn(ii), Fe(ii), Cd(ii) or Co(ii)).^12^,^21^,^23^

Most of the observed signals were relatively broad (I, II, Fig. 2B) and/or were composites of more than one peak (IV, iii, iv, Fig. 2B), with the sharpest being III (Fig. 2A), *i.e.* that assigned as corresponding to the proposed catalytically preferred di-Zn(ii) form. It is possible that the composite signals assigned to the mono-Cd(ii) and mono-Zn(ii) species (iii and IV, respectively Fig. 2), reflect binding in the two distinct (DCH or 3H site) metal ion ligating sites, though they may also reflect different protein conformations or trifluoromethyl ketone hydration states.^13^,^24^ The metal ions in the NDM-1* mono-Cd(ii) or di-Cd(ii) complex(es) were apparently readily substituted by Zn(ii) ions, whereas the signals corresponding to the NDM-1*-di-Zn(ii) complex remained stable on titration with Cd(ii) (Fig. S9A†). These observations imply binding of Cd(ii) is less efficient compared to that of Zn(ii), in accord with the reported lower affinity of Cd(ii) compared to Zn(ii) for NDM-1.^25^ The addition of Mn(ii) ions to the 1*-di-Zn(ii) complex led to broadening of the signals likely due to the paramagnetic effects of Mn(ii) (Fig. S9B†).

With Zn(ii) titrations only minor differences in the observed signals were observed at pH 7.5, 6.5, or 5.5 (Fig. S1–S4†). Cd(ii) binding was apparently more affected by pH, with least efficient binding at pH 7.5, perhaps reflecting less efficient binding of Cd(ii) compared to Zn(ii) (Fig. S8†). Differences in peak shape and chemical shift were also observed with Cd(ii) addition at different pHs (Fig. S5–S7†).

We then investigated the time dependence of Zn(ii) and Cd(ii) binding to NDM-1* (Fig. 3). Addition of 1 equivalent of Zn(ii) to apo NDM-1* manifests initial observation of signals assigned to the di- and mono-Zn(ii) species (III and IV, respectively, Fig. 3), which apparently slowly equilibrate towards the latter, though both species were present on extended incubation (Fig. 3A). A similar observation was observed when 2 equivalents of Zn(ii) were used, with peak III being relatively more intense (Fig. 3B). With 1 equivalent of Cd(ii), the peak set assigned as arising from the mono-Cd(ii) species predominates (iii, Fig. 3C). With 2 equivalents of Cd(ii), both mono- and di-Cd(ii) species were observed (iii and iv, respectively, Fig. 3), with the ratio of these two species being approximately stable over the duration of the analysis (Fig. 3D).

**Fig. 3:**
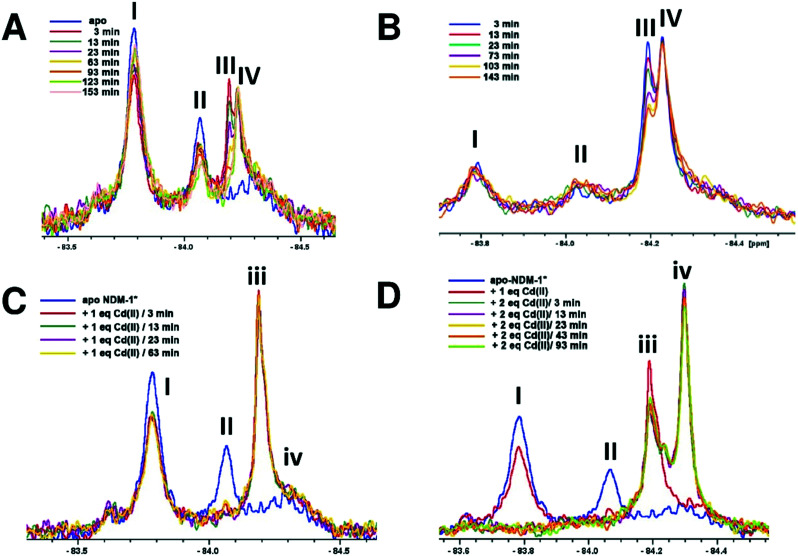
^19^F NMR analysis of the time dependence of Zn(ii) and Cd(ii) binding to NDM-1*. NDM-1* was incubated with (A) 1 equiv. of Zn(ii), (B) 2 equiv. of Zn(ii), (C) 1 equiv. Cd(ii), or (D) 2 equiv. Cd(ii), at pH 7.5, and the reactions monitored over time.

Because the ^19^F NMR (and other) results imply NDM-1 behaves differently with respect to different metal ions (Cd(ii) *vs.* Zn(ii)) in accord with the literature,^26^ we investigated how metallation is affected by an inhibitor, choosing to work with l-captopril, which is a well-studied NDM-1* inhibitor (*K*_D_ = 17 μM)^20^ that binds to the active site of B1 and other MBLs in a metal ion dependent manner.^20^,^21^ l-Captopril was shown to inhibit both Cd(ii) and Zn(ii) forms of NDM-1 under our standard assay conditions^11^ (IC_50_s: 1.3 and 3.8 μM with Zn(ii) and Cd(ii), respectively).

When apo NDM-1* was incubated with 2 equivalents of l-captopril no substantial differences in the ^19^F NMR spectrum were observed, consistent with a requirement for Zn(ii) in inhibitor binding (Fig. 4A). When 1 equivalent of Zn(ii) was added to a pre-incubated solution of apo NDM-1* and l-captopril, we observed formation of signals corresponding to the NDM-1*-di-Zn(ii)-l-captopril complex (V, Fig. 4), as assigned in previous work^20^ along with a decrease in the apo NDM-1* peak (I, Fig. 4A).^21^ A signal was observed at ∼*δ* –84.2 (VI, Fig. 4A); this may reflect a mono- or di-Zn(ii) species or a mixture of these, though it may reflect different protein conformations/other reaction. Over 2 hours a small decrease in the assigned NDM-1*-di-Zn(ii)-l-captopril (V) complex peak, with a corresponding increase in peak VI, was observed (Fig. 4C).

**Fig. 4:**
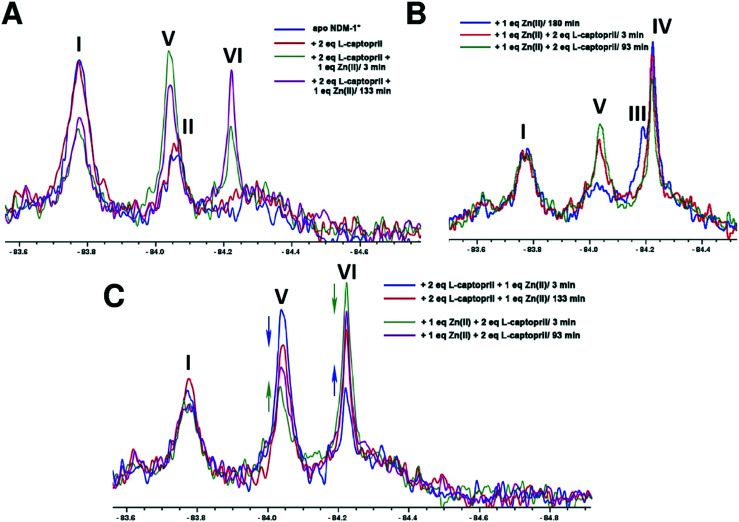
^19^F NMR analysis of the binding of l-captopril to NDM-1*. (A) Apo-NDM-1* was incubated with 2 equiv. of l-captopril, then treated with 1 equiv. of Zn(ii), (B) apo-NDM-1* was incubated with 1 equiv. of Zn(ii), then treated with 2 equiv. of l-captopril, (C) comparison of NMR spectra for the NDM-1*-Zn(ii)-l-captopril complex after incubation with the shown ratios of l-captopril or Zn(ii) ions.

When NDM-1* that had been preincubated with 1 equivalent of Zn(ii) was treated with 2 equivalents of l-captopril, formation of the NDM-1*-di-Zn(ii)-l-captopril complex (V, Fig. 4B) was observed, with a decrease in the NDM-1*-di-Zn(ii) complex (III, Fig. 4B), consistent with binding of l-captopril to the di-Zn(ii)-NDM-1* species (Fig. 4B). No change in the apo NDM-1* peak (I) was observed, confirming inhibitor binding requires (a) metal ion(s) (Fig. 4B). Samples that had been incubated with (i) 2 equivalents of l-captopril, then titrated with Zn(ii) or (ii) incubated with 1 equiv. Zn(ii), then titrated with l-captopril, gave similar spectra (Fig. 4C).

An interesting observation arose when an excess of l-captopril was added to NDM-1* preincubated with 1 equiv. of Zn(ii) (Fig. 5A). In addition to observation of the NDM-1*-di-Zn(ii)-l-captopril complex (V) and loss of the two signals corresponding to the mono- and di-Zn(ii)-NDM-1* species^20^ (Fig. 5, IV and III), the signal with a chemical shift between the assigned mono- and di-Zn(ii)-NDM-1* bound species emerged (Fig. 5B, VI). These observations support the proposal that binding of l-captopril requires (or at least is preferred by) the di-metallated form of NDM-1. Moreover, although other explanations are possible, the observations regarding peak VI, suggest the exchange rate between the assigned NDM-1*-mono-Zn(ii) and NDM-1*-di-Zn(ii) forms may be influenced by the presence of the l-captopril inhibitor in a concentration dependent manner.

**Fig. 5:**
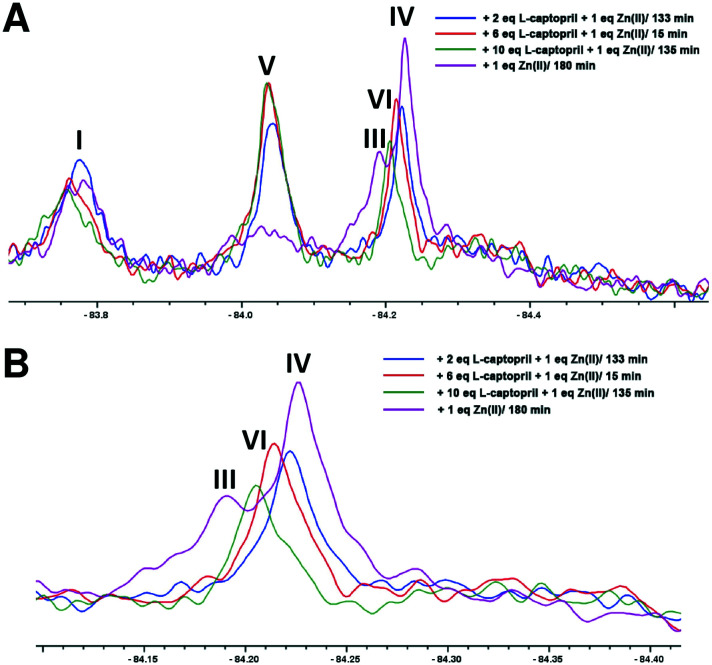
Binding of l-captopril to NDM-1*. (A) 1 equiv. Zn(ii) was incubated with apo-NDM-1* to form the NDM-1*-Zn(ii) complex, then l-captopril (2, 6 or 10 equiv.) was added to the NDM-1*-Zn(ii) complex, (B) close-up of the assigned NDM-1*-Zn(ii) complex peaks in the region of *δ* –84.2 on titration with l-captopril.

## Conclusions

The combined results imply that ^19^F protein observed NMR is a useful method for monitoring metal ion binding, as exemplified with studies on NDM-1. The generality of the approach should emerge as the method is tested within the context of studies on other metallo-enzymes. The overall ^19^F observations suggest a preference for binding of two Zn(ii) ions by NDM-1*, at least with excess Zn(ii). This conclusion is consistent with previous proposals for NDM-1, based on UV-VIS, ^1^H NMR, EPR, EXAFS, and X-ray, kinetic, and (our) native mass spectrometric studies, validating the ^19^F method (Fig. S10 and S11†).^24^,^27^ The ^19^F method also implies differences in the interactions of NDM-1* with different metal ions, as exemplified with studies with Zn(ii) and Cd(ii) binding to NDM-1*; again the ^19^F results are consistent with literature reports.^8^,^10^,^26^ Notably, the results reported here suggest that the mono- and di-zinc ion forms of NDM-1* do not differ greatly in their stability (Fig. S1†), as revealed by titration and time course studies. Thus, due consideration should be given as to the most relevant form of NDM-1 *in vivo*. A caveat of the ^19^F method is the assumption that metal ion binding is the same for the wildtype and the ^19^F labelled protein; this may not always be the case, but at least for NDM-1/NDM-1* this does not appear to be a major concern. It is also notable that protein observed ^19^F NMR of NDM-1* can be used to probe events at the metal-ion binding site, despite the ^19^F-label being >5 Å away from it (Fig. 1). Thus, changes in the ^19^F spectra may reflect differences in conformation/solvation/hydration rather than direct interactions of the ^19^F-labelled residue with the metal ion binding centre/ligand, as precedented in other applications of protein observed ^19^F NMR.^19^

^19^F NMR is suited for studying the roles of metal ions in inhibitor binding, as exemplified with our work on l-captopril and NDM-1*, the results of which are consistent with reports concerning inhibitor binding to the di-Zn(ii)-NDM-1.^20^ The sensitivity of the ^19^F NMR method can enable observation of even small perturbations in the chemical environment of the ^19^F label, including in a time dependent manner and when the binding event is not occurring in the immediate vicinity of the label.^28^ Although the interpretations should be regarded as preliminary, the observations suggest that the exchange rate between NDM-1*-mono-Zn(ii) and NDM-1*-di-Zn(ii) might be influenced by the presence of an inhibitor, illustrating the use of ^19^F NMR in mechanistic medicinal chemistry on metallo-enzymes.

## Conflicts of interest

There are no conflicts to declare.

## Supplementary Material

Supplementary information
