# Supplementary material for: Monitoring protein-metal binding by 19F NMR – a case study with the New Delhi metallo-β-lactamase 1
Source: RSC Med Chem. 2020 Feb 21;11(3):387–91. doi: 10.1039/c9md00416e (PMC7484990; doi:10.1039/c9md00416e)
Supplement: Supplementary file 1 [file MD-011-C9MD00416E-s001.pdf]

Electronic Supplementary Material (ESI) for RSC Medicinal Chemistry.
This journal is © The Royal Society of Chemistry 2020

Supporting Information

Monitoring protein-metal binding by ^{19}F NMR – A case study with the New Delhi metallo-β-lactamase 1

Anna M. Rydzik[a‡§], Jürgen Brem[a‡], Shane A. Chandler[a], Justin L.P. Benesch[a], Timothy D. W. Claridge[a], and Christopher J. Schofield[a]*

[a]*The Department of Chemistry, University of Oxford, 12 Mansfield Road, Oxford, OX1 3TA, United Kingdom.*

‡*These authors contributed equally to this work.*

§*Current address: Research and Early Development, Respiratory, Inflammation and Autoimmunity, BioPharmaceuticals R&D, AstraZeneca, Gothenburg, Sweden.*

** Correspondence to: christopher.schofield@chem.ox.ac.uk*

Experimental Details

Chemicals were from Sigma-Aldrich, or as stated otherwise. The purity of $ZnSO_4$, $CdSO_4$ and $MnSO_4$, used in titrations was ≥99.99%. Recombinant NDM-1 M67C (> 95% purity by SDS PAGE analysis) was produced and labelled using bromotrifluoroacetone (BFA) via a reported protocol.[1] Apo-NDM-1* was generated using a reported protocol.[2] In brief, di-Zn(II) NDM-1* solutions were dialysed against three changes of > 100 volumes of the original solution of an EDTA-containing solution (50 mM HEPES pH 7.5, 200 mM NaCl, 20 mM EDTA, 2 mM TCEP·HCl). EDTA was removed by a second dialysis of three changes of > 100 volumes of a metal-free solution (50 mM HEPES pH 7.5, 200 mM NaCl, 2 mM TCEP·HCl, Chelex 100). All dialyses were carried out at 4 °C using 2K Slide-A-Lyzer® Dialysis Cassettes (Thermo Scientific). Samples containing apo NDM-1 were stored at -80 °C; prior to NMR measurement they were thawed on ice and buffer exchanged using a Bio-Spin® 6 column (Bio-Rad) to give the final buffer (HEPES/MES buffer (50 mM, pH 7.5, 6.5 or 5.5 respectively) which was supplemented with 200 mM NaCl and 10% D_2O.

^{19}F NMR measurements

^{19}F NMR measurements were conducted at 298 K using a Bruker AVIII HD 600 spectrometer equipped with a 5mm z-gradient broadband Prodigy N_2-cryoprobe constructed with no fluorine background operating at 298K. Samples contained apo NDM-1* (60 µM) in HEPES buffer (50 mM, pH 7.5) supplemented with 200 mM NaCl and 10% D_2O in 5 mm diameter NMR tubes (Norell). Spectra were recorded typically using 128 scans. Data were processed using TopSpin 3.1 software (Bruker) and were referenced to an internal trifluoroacetic acid (TFA) standard (δ - 75.45 ppm) as reported.[1]

Non-denaturing Mass Spectrometry measurements

Protein samples were buffer exchanged into a 200 mM NH_4OAc aqueous solution using a Bio-Spin spin column (Bio-Rad, Hercules, USA). Samples were further diluted with NH_4OAc (Sigma-Aldrich, Gillingham, UK) to 5 µM to which a solution of zinc acetate was added at the required ratio, resulting in a final protein concentration of 2.5 µM. The protein-zinc solution was incubated on ice for 1.5 hours prior to analysis. Non-denaturing mass spectrometry was performed in the positive ion mode on the incubated samples as described,[3] using a nano-electrospray source and gold-coated borosilicate capillaries. Capillaries were prepared in-house using a model P-97(Sutter Instruments) capillary puller and a sputter coater (Polaron, Newhaven, United Kingdom). Ions were introduced into a Q Exactive hybrid quadrupole-Orbitrap mass spectrometer (Thermo Fisher Scientific, Bremen Germany) modified for the transmission, selection and detection of high mass ions (Rose et al., 2012).[4] All spectra were

acquired in the ‘Native Mode’ with no in-source activation nor HCD activation voltage applied. The Orbitrap analyser was operated at a nominal resolution of 17,500 and the AGC target was $1x10^6$. The collision gas was nitrogen and UHV pressure was maintained at approximately $1x10^{-9}$ mbar. Spectra were acquired with 10 microscans, averaged over a minimum of 100 scans and with a noise level parameter set to 3. Each protein-zinc analysis was performed in duplicate or triplicate from separate zinc incubations. Spectra were acquired and then averaged using Thermo Scientific Xcalibur 2.1.

Table

Table S1. Reported data on the kinetic properties of labeled and unlabeled NDM-1 variants.[1]

**Enzyme**		**K_M [μM]**	**k_{cat} [s^{-1}]**
NDM-1	Meropenem	77±4	235±5.6
NDM-1*	Meropenem	55±9	68±4
NDM-1	Nitrocefin	9±2	25±2
NDM-1*	Nitrocefin	6±1	23±1

Figures

A

B

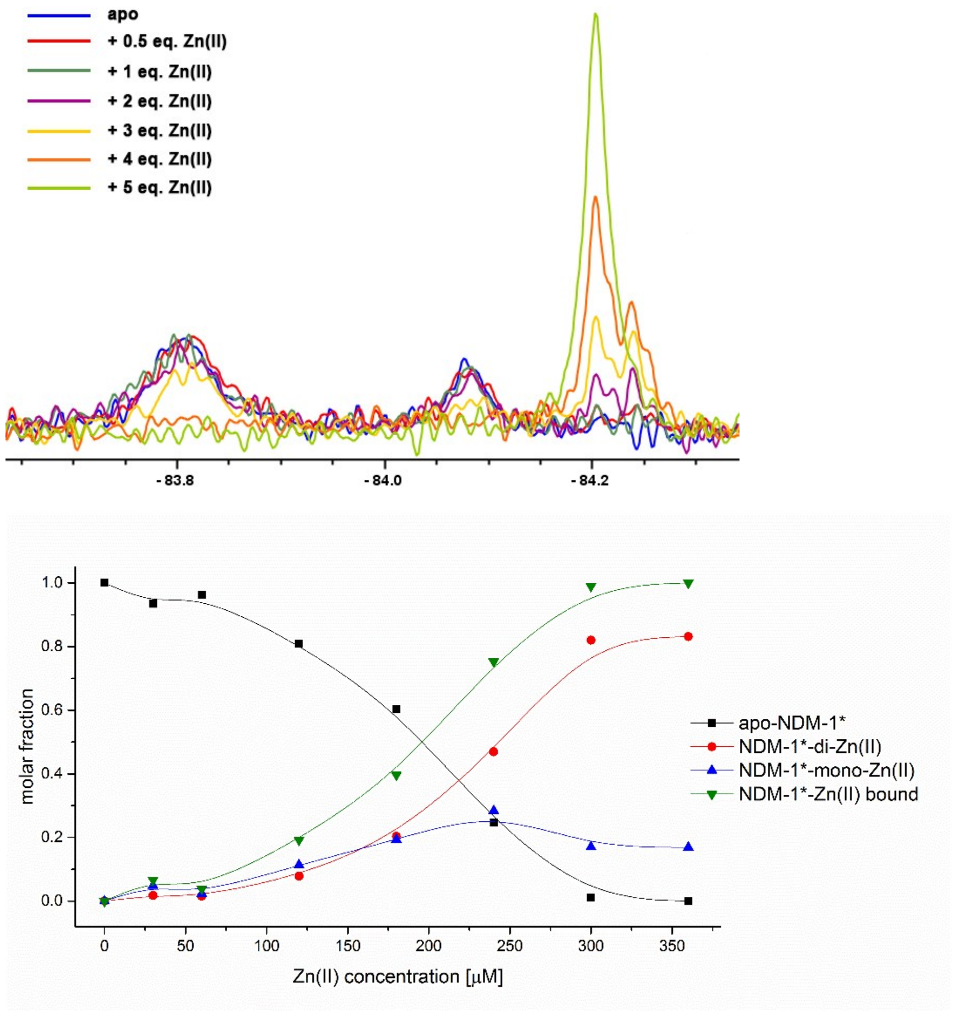

Figure S1. Formation of the assigned mono-Zn(II) and di-Zn(II) NDM-1* (60 μM) bound species at pH 7.5 as monitored by ^{19}F NMR, following addition of different Zn(II) equivalents. See Experimental Section for methods.

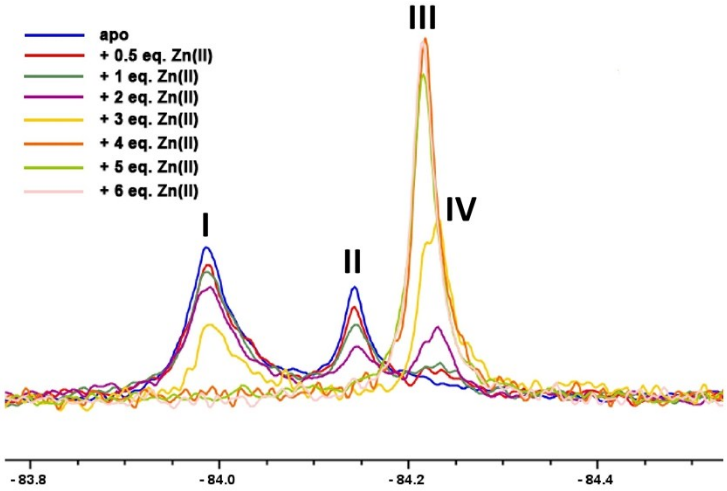

Figure S2. Titration of apo-NDM-1* (60 μM) with Zn(II) at pH 6.5. See Experimental Section for methods.

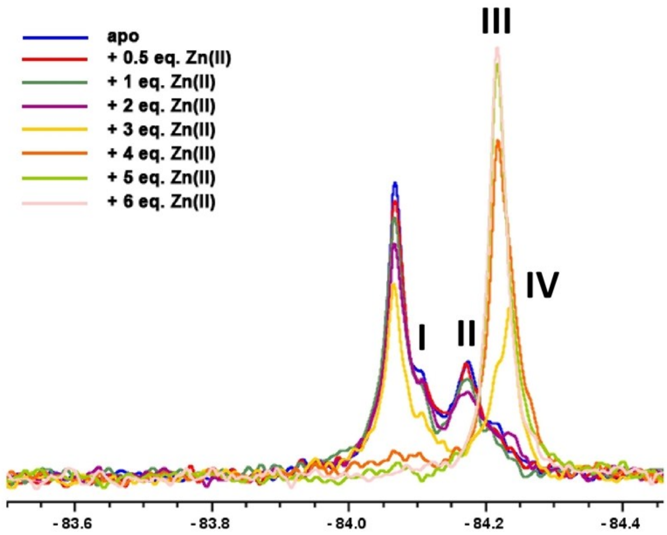

Figure S3. Titration of apo-NDM-1* (60 μM) with Zn(II) at pH 5.5. See Experimental Section for methods.

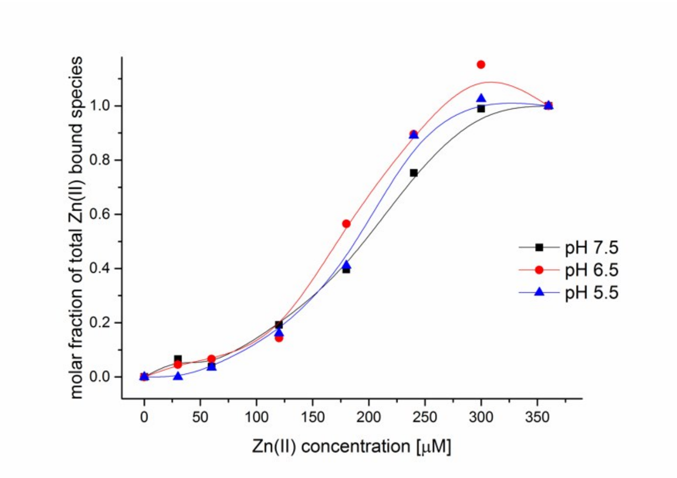

Figure S4. Summary of Zn(II) binding to apo-NDM-1* (60 μM) at different pH values.

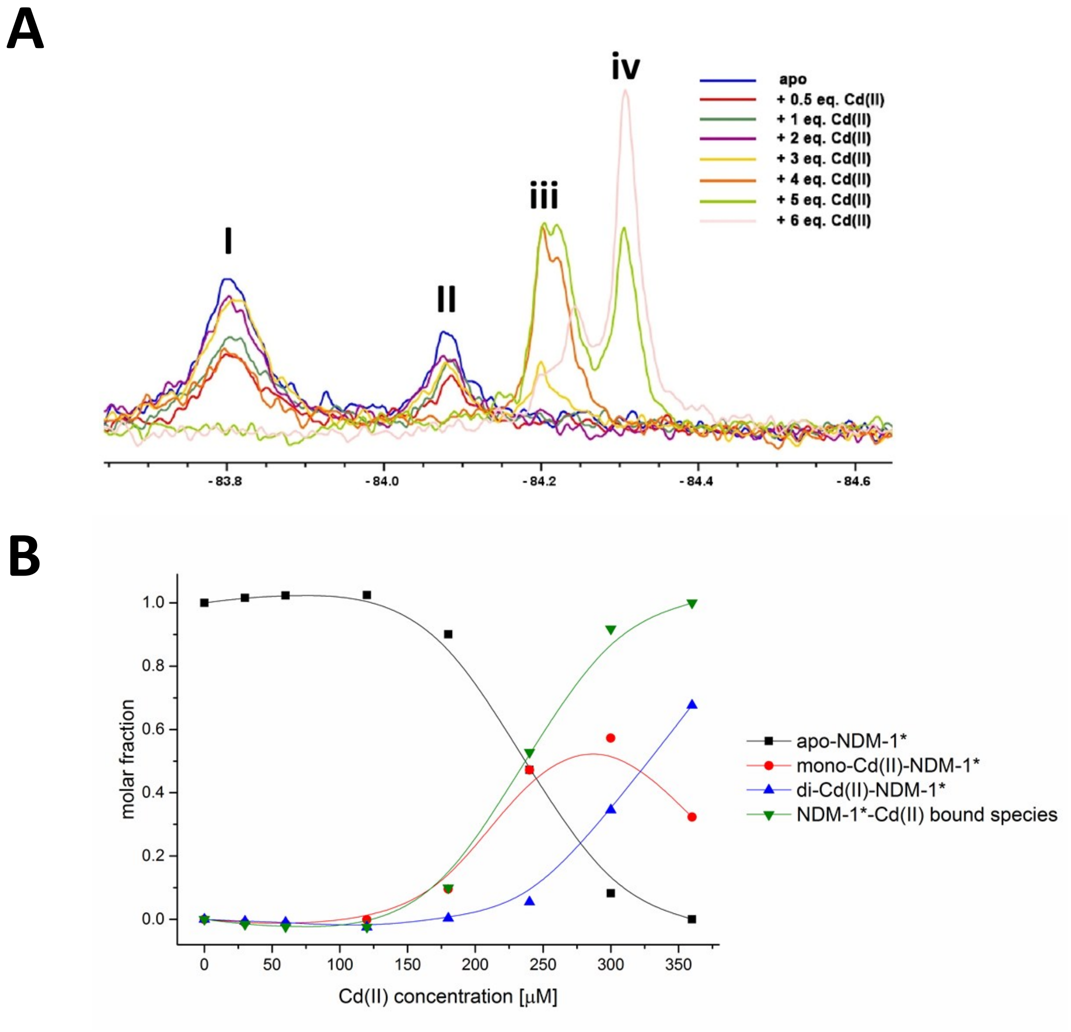

Figure S5. Formation of the assigned mono-Cd(II) and di-Cd(II) NDM-1* (60 μM NDM-1*) bound species at pH 7.5 as monitored by ^{19}F NMR, following addition of different Cd(II) equivalents. See Experimental Section for methods.

A

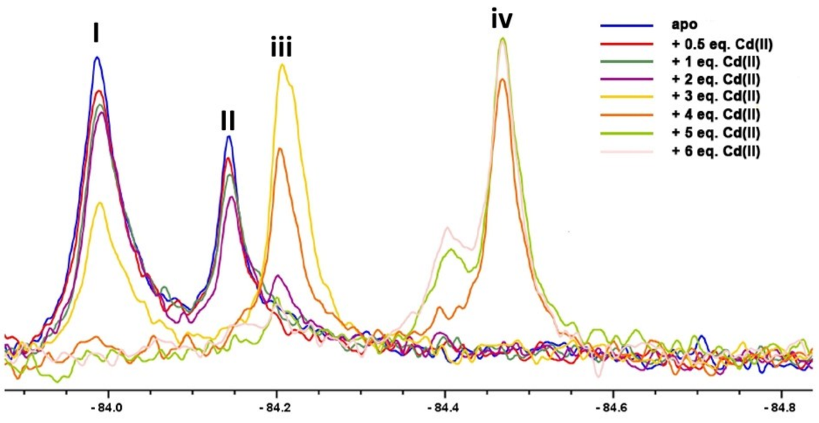

B

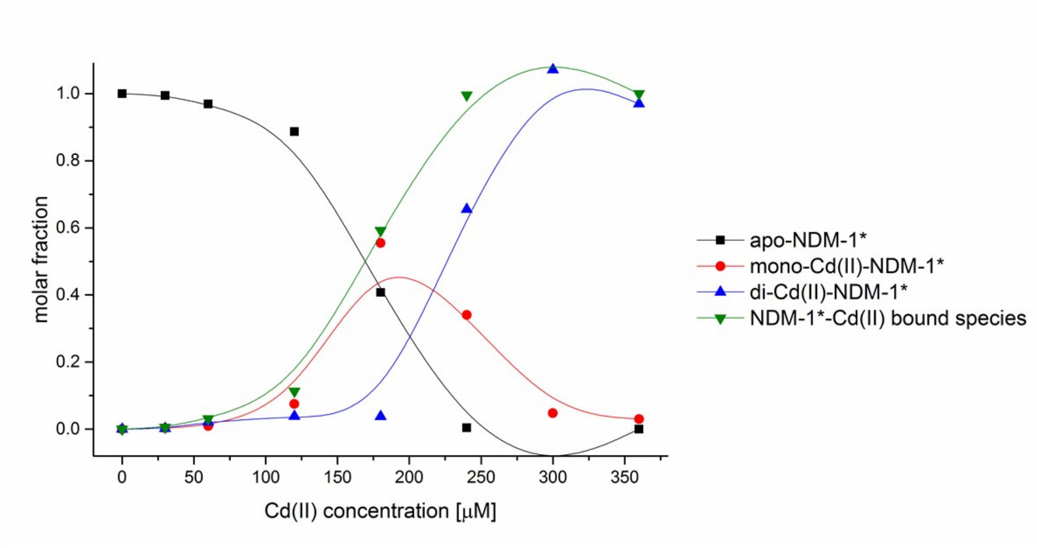

Figure S6. Formation of the assigned mono-Cd(II) and di-Cd(II) NDM-1* (60 μM NDM-1*) bound species at pH 6.5 as monitored by ^{19}F NMR. See Experimental Section for methods.

A

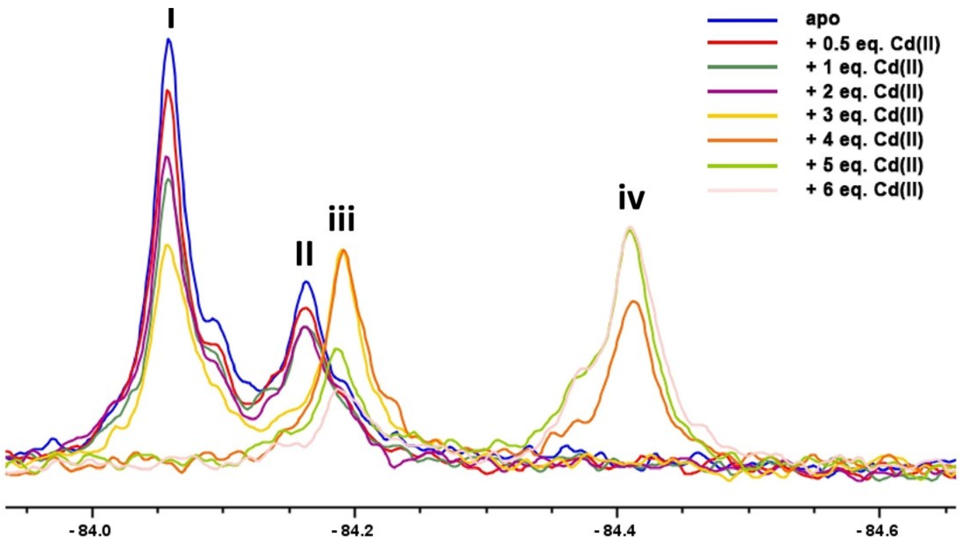

B

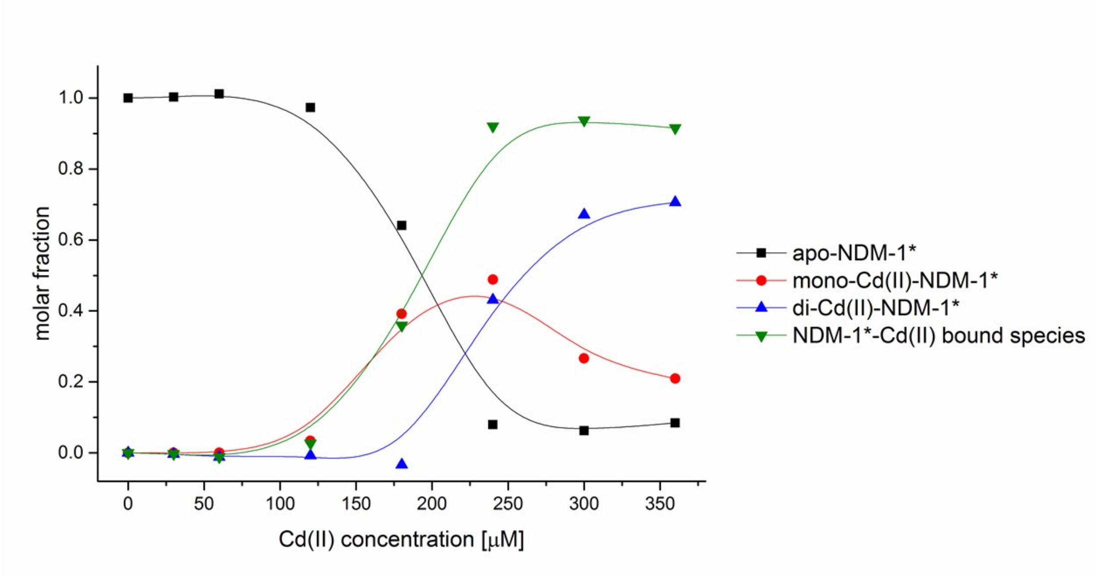

Figure S7. Formation of mono-Cd(II) and di-Cd(II) NDM-1* (60 μM NDM-1*) bound species at pH 5.5 as monitored by ^{19}F NMR. See Experimental Section for methods.

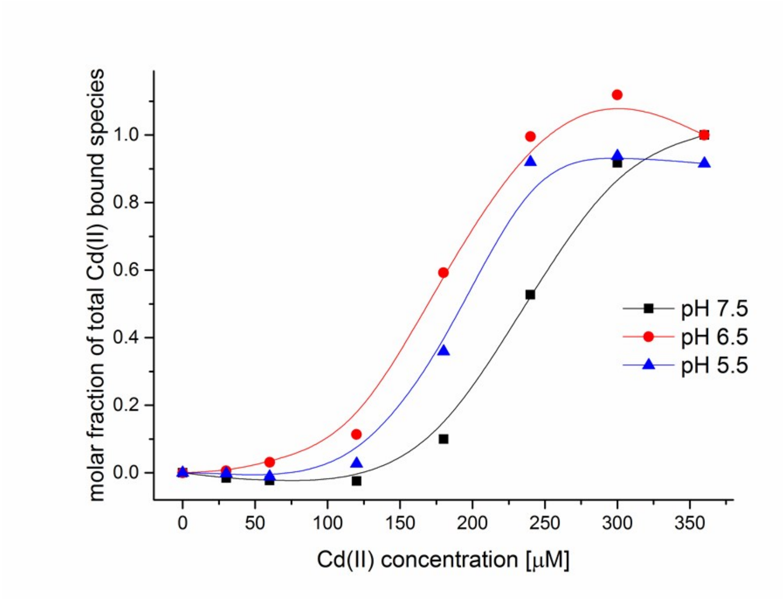

Figure S8. Summary of ^{19}F NMR results comparing Cd(II) binding to apo-NDM-1* (60 μM NDM-1*) at different pH values.

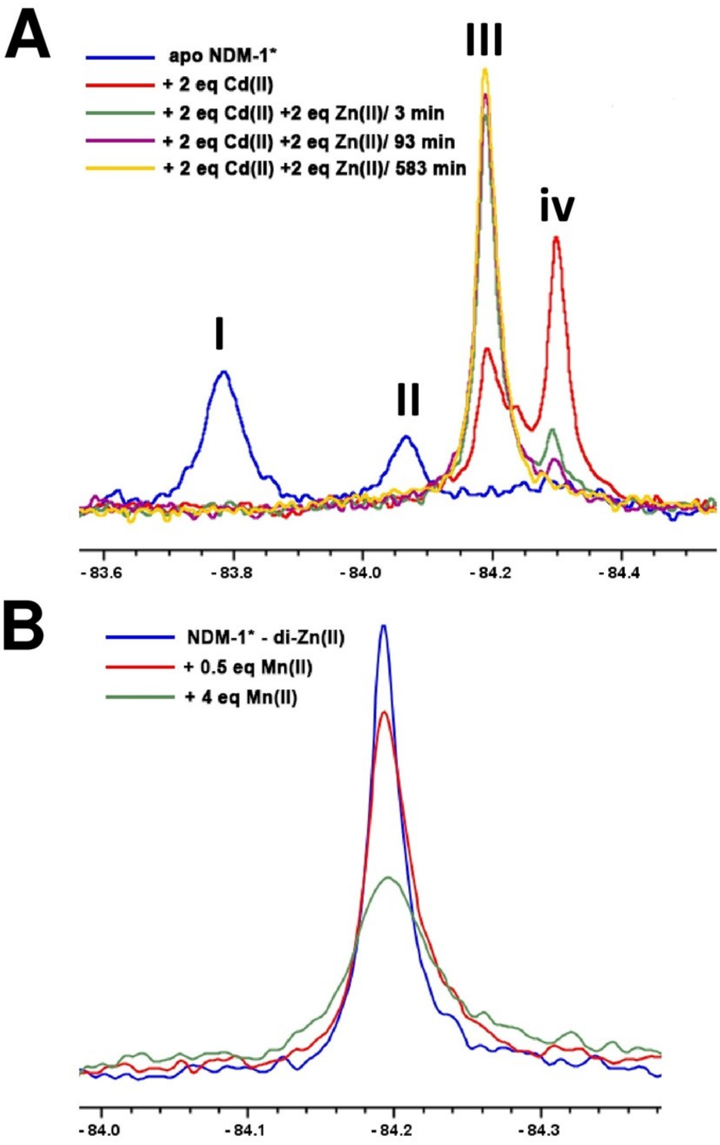

Figure S9. **Metal displacement experiments.** (A) The assigned NDM-1*-Cd(II) complex (red) (60 μM) was treated with 2 equiv. of Zn(II) (green, purple, orange). The results imply Zn(II) ions bind with higher affinity than Cd(II) ions. Addition of 2 equiv. of Zn(II) to the mixture of NDM-1* equilibrated with 2 equiv. of Cd(II) yields resonances corresponding to the assigned di-Zn(II) bound species with loss of the assigned di-Cd(II) species. Due to overlap of signals it is uncertain if any mono-Cd(II) bound species are present. (B) NDM-1*-Zn(II) complex (60 μM) treated with 0.5 and 4 equiv. of Mn(II). On titration with Mn(II) broadening of the resonances corresponding to the assigned di-NDM-1*-Zn(II) species is observed likely due to the paramagnetic nature of Mn(II). See Experimental Section for methods.

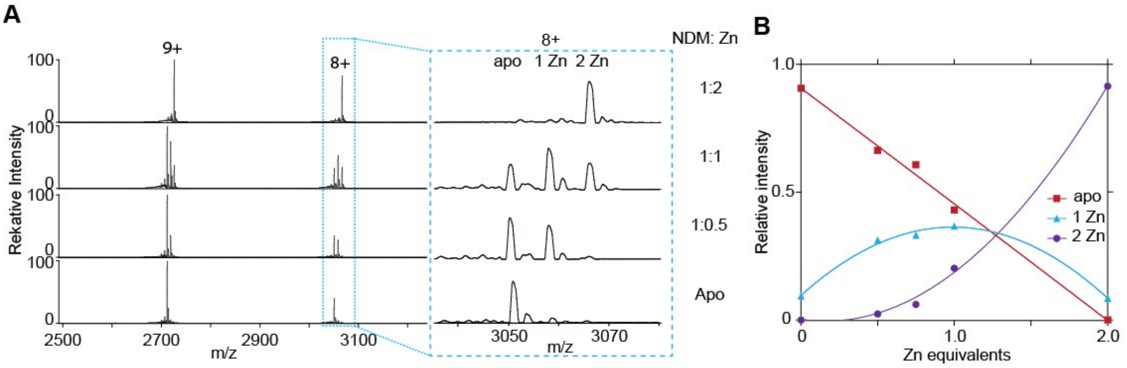

Figure S10. Investigation of zinc binding stoichiometry to NDM-1 (2.5 μM) using non-denaturing mass spectrometry. (A) Mass spectra of NDM-1 show up to two zinc bound to the apo protein after titration and incubation with $Zn(OAc)_2$ for 1.5 hours. (B) Occupancy of zinc binding sites throughout the titration using data averaged across both observable charge states.

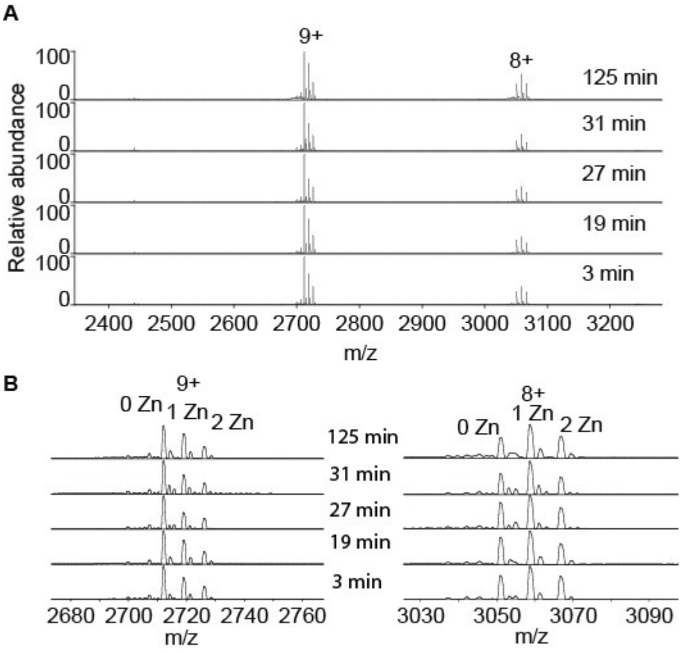

Figure S11. Non-denaturing mass spectra of NDM-1 (2.5 μM) incubated with one molar equivalent of $Zn(OAc)_2$ for 3-125 minutes. The spectra show no evidence for time dependency on the binding stoichiometry. (A) The relative abundance of the 8+ and 9+ charge states does not change. (B) The ratio of apo-, mono- and di-Zn(II) forms remains constant over the time course of incubation.

References

1. A. M. Rydzik, J. Brem, S. S. van Berkel, I. Pfeffer, A. Makena, T. D. Claridge and C. J. Schofield, *Angew. Chem. Int. Ed. Engl.*, 2014, **53**, 3129-3133.
2. S. T. Cahill, H. Tarhonskaya, A. M. Rydzik, E. Flashman, M. A. McDonough, C. J. Schofield and J. Brem, *J. Inorg. Biochem.*, 2016, **163**, 185-193.
3. F. D. Kondrat, W. B. Struwe and J. L. Benesch, *Methods. Mol. Biol.*, 2015, **1261**, 349-371.
4. R. J. Rose, E. Damoc, E. Denisov, A. Makarov and A. J. Heck, *Nat. Methods.*, 2012, **9**, 1084-1086.
